# Immunosuppressive strategies in face and hand transplantation: a comprehensive systematic review of current therapy regimens and outcomes

**DOI:** 10.3389/frtra.2024.1366243

**Published:** 2024-03-06

**Authors:** Lioba Huelsboemer, Sam Boroumand, Alejandro Kochen, Alna Dony, Jake Moscarelli, Sacha C. Hauc, Viola A. Stögner, Richard N. Formica, Bohdan Pomahac, Martin Kauke-Navarro

**Affiliations:** ^1^ Division of Reconstructive and Plastic Surgery, Yale School of Medicine, New Haven, CT, United States; ^2^ Regenerative Wound Healing Center, Yale School of Medicine, New Haven, CT, United States; ^3^ School of Medicine, University of Leeds, Leeds, United Kingdom; ^4^ Department of Medicine, Section of Nephrology and Transplantation, Yale School of Medicine, New Haven, CT, United States

**Keywords:** face transplant, hand transplant, vascularized composite allograft (VCA), immunosuppressive therapy, outcome

## Abstract

**Background:**

Recipients of Vascularized Composite Allotransplants require effective immunosuppressive therapy to prevent graft rejection. This systematic review summarizes the current body of literature on immunosuppressive regimens used in face and hand transplants while summarizing their outcome in terms of rejection, renal failure, and infections.

**Methods:**

A systematic search of electronic databases was conducted to identify relevant studies from 1998 until July 1st, 2023. We included all studies that discussed immunosuppressive strategies in face and hand transplant recipients according to PRISMA.

**Results:**

The standard triple maintenance therapy was mostly adjusted due to nephrotoxicity or high incidence of rejection. The most common alternative treatments utilized were sirolimus (25/91; 27.5%) or everolimus (9/91; 9.9%) following hand- and photophoresis (7/45; 15.6%), sirolimus (5/45; 11.1%) or belatacept (1/45; 2.2%) following face transplantation. Episodes of rejection were reported in 60 (65.9%) of hand- and 33 (73%) of face transplant patients respectively. Graft loss of 12 (13.2%) hand and 4 (8.9%) face transplants was reported. Clinical CMV infection was observed in 6 (6.6%) hand and 7 (15.5%) face transplant recipients.

**Conclusions:**

Based on the herein presented data, facial grafts exhibited a heightened incidence of rejection episodes and CMV infections. Facial mucosa adds complexity to the immunological graft composition highlighting the need of individualized immunosuppressive regimens and further research.

## Introduction

Vascularized Composite Allotransplantation (VCA) is currently the highest available reconstructive option on the reconstructive ladder as it uniquely offers both functional (motor, sensory) and aesthetic reconstruction at the same time. A total of 300 VCAs including 148 upper extremity, 80 uterus, 48 face, 46 abdominal wall, five penis, and two lower extremity transplantations have been performed worldwide (1–5). In contrast, about 25,000 kidney transplantations have been performed in the United States in 2022 alone (6). Major obstacles such as short ischemia time tolerance of muscles and acute and chronic rejection prevent VCA from becoming a more widely utilized reconstructive procedure (7, 8). In contrast to solid organ transplantation (SOT), VCA is not categorized as a life-saving procedure; instead, it is regarded as a life-giving intervention. Therefore, the side effects of long-term immunosuppressive therapy (for example, cancer development and opportunistic infections) have so far been difficult to reconcile with the benefits of VCA. Due to the lack of longer-term follow up and smaller number of patients, the risk-benefit ratios of VCAs has yet to be formally defined. Immunosuppressive (IS) regimens have largely been adopted from SOT. Induction is often done with antithymocyte globulin (T cell depleting drug) or alternatively monoclonal antibodies such as Basiliximab or Alemtuzumab, followed by a triple maintenance therapy of Tacrolimus, Mycophenolate Mofetil (MMF), and steroids (9). Even though the different levels of immunogenicity in skin and kidney are well described (e.g., by Moseley et al.), with skin being more immunogenic than kidneys, VCA IS regimens are largely based on experience in SOT (10). Additionally, research in the field of face transplantation revealed that mucosa might be more immunogenic than skin and may reject at a higher frequency (11–13). These findings could lead to the assumption that VCA and especially face transplant patients must be treated differently as the immunogenicity of skin and mucosa seem to differ from solid organs. New treatment options, improved understanding of the molecular rejection mechanisms in skin and mucosa, and standardized guidelines for VCA are urgently needed to not only reduce toxic long term side effects of immunosuppressants but to also allow more targeted IS in VCA recipients. Current research in animal models mostly focuses on the principle of tolerance induction by directly targeting the recipient's immune system through cellular or pharmaceutical approaches (9, 14, 15). As new therapeutics are not yet available for humans and standardized guidelines are non-existent, this systematic review aims to provide a comprehensive overview of current treatment regimen in humans following VCA to give an update on treatment options worldwide.

## Methods

### Literature search

We conducted a systematic review of manuscripts listed in PubMed, MEDLINE, and Embase databases following the “preferred reporting items for systematic reviews and meta-analysis” (PRISMA) guidelines. The search strategy included both medical subject headings (MeSH) and directly quoted keywords relating to the following two concepts: VCA and immunosuppression treatments. We assessed outcomes such as graft survival, rejection rates, and complications in face and hand transplant recipients. Subgroup analyses investigated the impact of immunosuppression on face or hand or hand and face transplantation. This search strategy was adapted across each of the databases according to their individual requirements. The full electronic search strategies for each database are shown in the Supplementary Figure S1. We included all studies from inception of each database to the search date of July 1st, 2023. Utilizing a two-step approach, S.B. and A.K. independently examined the results of the search criteria for titles and abstracts. Any discrepancies in study inclusion/exclusion were resolved by a third reviewer (L.H.). Subsequently, both S.B. and A.K. performed full-text review of all included studies.

### Study selection criteria

Following query of the search results, manuscripts were automatically excluded (utilizing database result filters) if they did not meet the following criteria: Full-Text availability, Human studies, and English language. After evaluating the remaining abstracts/titles, all editorials, reviews, commentaries, and conference abstracts were additionally excluded leaving primarily original articles and case reports. All remaining manuscripts received full-text evaluation to identify specific elements including: the VCA center responsible for the study, the type of VCA in the study (e.g., hand vs. face), the number of patients included, the immunosuppression regimens utilized, and any noted complications/effects of the immunosuppressive treatments based on systemic categories. Rejection in skin or mucosa (face transplant) or skin (hand transplant) was included if reported as graded >1 according to Banff Classification (T-cell mediated rejection). Clinical CMV infection was defined as serum positive plus clinical symptoms while CMV viremia was defined as isolated DNAemia without evidence of end-organ damage (16). References of included studies were reviewed for additional studies. All data extracted from included studies were independently entered by first author and year of publication into a Microsoft Excel worksheet by two reviewers (S.B. and A.K.) for tabulation and analysis. Disagreements between inputs were resolved by discussion and consensus from a third reviewer (L.H.).

## Results

### Search outcomes

**Figure 1:**
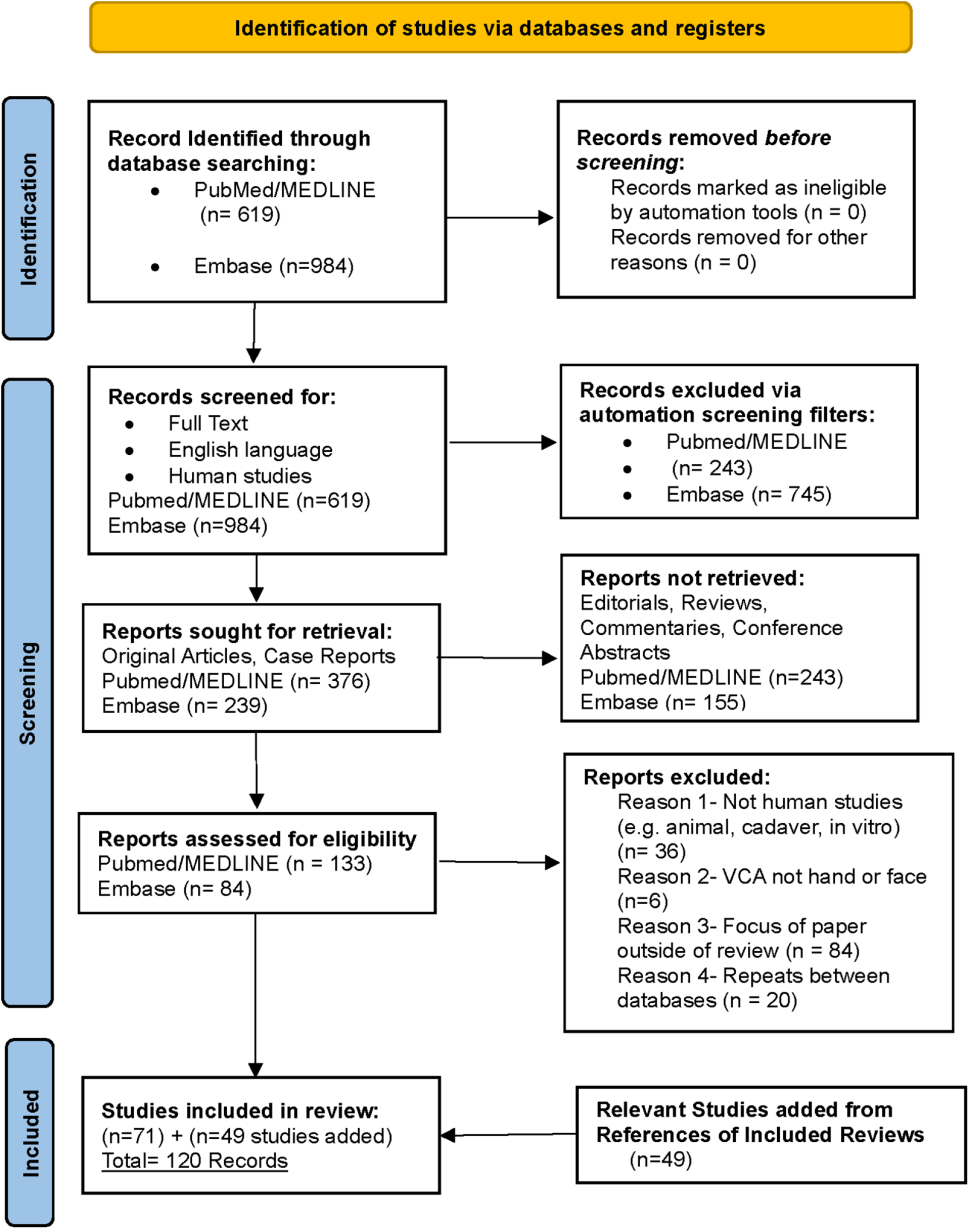
The search strategy according to the PRISMA guidelines yielded 120 articles that met all inclusion criteria. (Adapted from (17).

**Figure 2:**
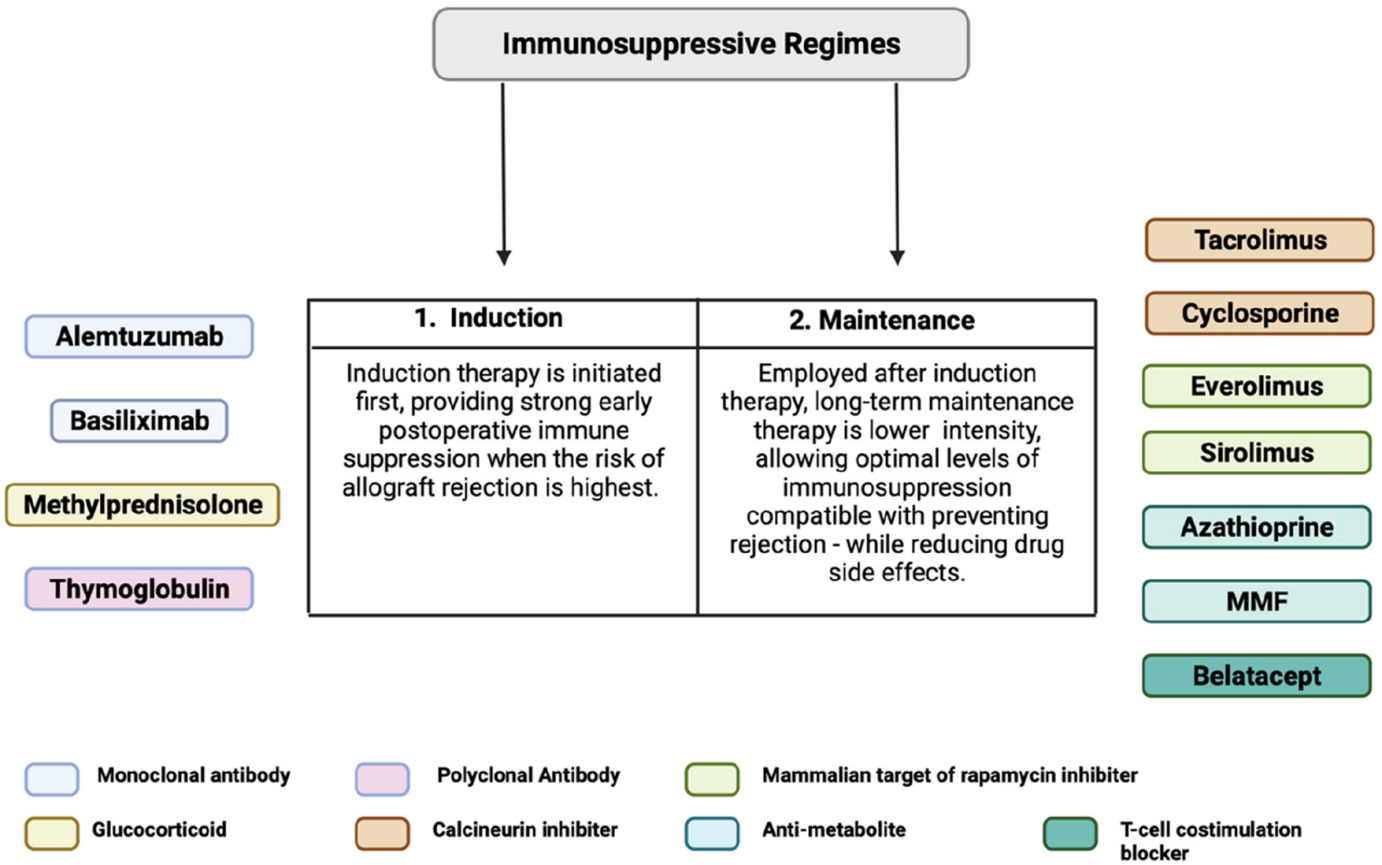
Immunosuppressive regimen model for VCA transplantation (18–20).

The search strategy yielded 120 articles that met all inclusion criteria (Figure 1). The articles were published between 1999 and 2023. 47 articles discussed immunosuppressive regimens (Figure 2) for face VCAs, 71 articles pertain to the immunosuppressive regimen of hand VCAs, and two articles pertain to those patients who have received both hand and face VCAs. In total the search strategy captured 45 patients of facial transplantation, 91 cases of hand transplantation, and 3 cases of combined face and hand transplantation. A breakdown of the immunosuppressive treatments and associated complications for each VCA can be identified in Tables 1–3. Associated complications were broken down into the following applicable categories: Rejection, Infectious, Renal, Metabolic, Deaths, Graft Loss, Hematologic, Malignancy, and Other.

**Table 1:** Immunosuppressive induction and maintenance regimens and significant outcomes reported across VCA facial transplantation centers*.*

Center	Number of patients	Immuno-suppressive induction THERAPIES utilized	Starting immuno-suppressive maintenance therapies utilized	Significant outcomes reported (per patient/timepoint)	Mucosa used as surveillance tool (y/n)	TCMR episodes (maximal reported follow up)	Reference
Ankara, Turkey	1- Partial	Methylprednisolone Thymoglobulin Tacrolimus MMF	Prednisolone Tacrolimus Azathioprine	Infection: Viral (CMV, clinical infection) at POM 5, Bacterial (Strep pyogenes sepsis, Retroperitoneal abscess), Fungal (Oral/Esophageal candidiasis) at POM 5 and POM 36 Renal: Renal dysfunction (POM 5), retroperitoneal abscess in left kidney (POM 36) Death: 56 months post-transplant (DIC & sepsis Streptococcus pyogenes) GI: Diarrhea POM 36 Metabolic: Weight loss, Vertebral Osteoporosis (POM 36) Hematologic: Neutropenia (POM 5) Other: Oronasal fistula (3rd week post-op until POM 11)	No	1 episode grade III on POD 26 (POM 56)	(21)
Cleveland, USA	1- Full 2- Partial	Methylprednisolone Thymoglobulin Tacrolimus MMF	Prednisone Tacrolimus MMF	Renal: Renal vein thrombosis in patient 3 at POM 49. Death: Patient 1 at 10.5 years post-transplant (DIC, sepsis due to worsening of chronic rejection) Hematologic: Neutropenia recurrent cycles in patient 1 at POD 21 and POM 6 Other: Patient 2 at POM 3 bilateral common femoral deep vein thromboses and brachial artery occlusion requiring thrombectomy, duodenal perforation 5 years post-transplant. Patient 3 was diagnosed with deep vein thrombosis at POM 1.	Patient 1: Grade III/IV on POD 47, 63, 77, 91 Patient 3: Grade II at POM 1&14	Patient 1: Rejection episodes POM 2, 1.5 and 4.5 years posttransplant. Chronic rejection 9 years posttransplant (POY 10.5) Patient 2: Rejection episodes at 1 year post-transplant, then 2 years, 2.5 years, and finally 6 years post-transplant for chronic recurring rejection requiring debridement and cadaveric skin grafting. (POY 6) Patient 3: Acute rejection episode POM 1, POY 3&4. (POY 4)	(22–25)
Boston/New Haven, USA	5- Full (including 1 retransplant) 4- Partial	Methylprednisolone Thymoglobulin MMF	Prednisone Tacrolimus MMF Patient 6: due to progressive nephrotoxicity, neurotoxicity and refractory CMV viremia, was switched to sirolimus POM 11, which led to significant lower extremity swelling, worsening renal function (proteinuria) and rejection, therefore was converted to belatacept POM14, Tacrolimus was added POM 18 due to rejection Low-dose IL-2	Infection: Patient 1: SSI POD8, infected sialocele POD 26, Cellulitis POY 2, 3 & 5, penile HSV POY 4, Granuloma POY 4, bacteremia POY 6, UTI POY 6, recurrent HCV POY 6, 8 & 9, Parotitis POY 9 Patient 2: Pneumonia POD 1, bacteremia POM 3, CMV gastritis POM 7, HSV POY 2 Patient 3: Pneumonia POD 1, VRE POM 1, Diarrhea (Clostridium diff.) POM 3&4, Norovirus POM 9, conjunctivitis POM 11, Sinusitis POY 3, blepharitis POY 4 Patient 4: Herpes Zoster POY 2, Cellulitis POY 3, Norovirus POY 3 Patient 5 (graft 1): CMV viremia at POM 6, oral candidiasis POM 8 & POY 2 Patient 6: CMV clinical infection refractory POY 1, Guillain-Barré syndrome POM 11 Patient 9: urinary tract infection, sepsis within POY 1 Patient 5 (retransplant): none reported Renal: progressive nephrotoxicity (Patient 6 POM 11) Graft Loss: Patient 4 (chronic rejection-retransplant of face 84 months after first) Death:Patient 1 POY 10 (hepatocellular carcinoma) Hematologic: Leukopenia Malignancy: Patient 1 hepatocellular carcinoma Other: Lower extremity edema and neurotoxicity (Patient 6 within POY 1)	Patient 9: Grade III POD 31 Patient 10: Grade III POM 4, 6	Patient 1: Grade II POM 43 & grade III POM 22, 42, 48, 58, 67, 74, 78, 83, 96 (POY 10) Patient 2: Grade II POM 1, 37 & grade III POM 18, 34, 47, 54 (POY 5) Patient 3: Grade II POM 2, 17, 47 & grade III POM 30, 59 (POY 5) Patient 4: Grade II POM 12 & grade III POM 21, 24, 38, 42, 53 (POY 5) Patient 5: Grade II POM 3 & grade III POM 8, 12, 17, 19, 26, 33, 38, 40, 42, 51, 53, 74, 78 (POM 78) Patient 6: Grade II&III at POM 3, 7, 8, 14 & 18 (POM 24) Patient 9: Grade II POM 31 (POM 31) Patient 10: Grade III POM 3 (POM 7)	(26–39)
Montreal, Canada	1- Partial	Methylprednisolone Thymoglobulin Tacrolimus MMF	Prednisone Tacrolimus MMF	Infection: Viral (CMV esophagitis POD 182), Bacterial (pneumonia, C. diff colitis, dacrocystitis), Fungal (mucormycosis) within POY 1 Renal: Elevated creatinine/AKI (not specified) Other: Perforated diverticulitis POD 335	No	None detected (POD 445)	(40, 41)
Helsinki, Finland	1- Full 1- Partial	Methylprednisolone Thymoglobulin Tacrolimus MMF	Corticosteroids Tacrolimus MMF	Infection: Viral (EBV) patient 1 POY 1 Renal: Elevated creatinine patient 1 through POY 2 Metabolic: Diabetes patient 1 through POM 3, Hypertension patient 1 POY 2 Other: Oronasal fistula patient 2 POM 1, Nasopalatinal fistula patient 1 through POM 14	No	No acute rejection episodes reported Patient 1 follow up POY 3 Patient 2 follow up POY 1	(42)
New York, USA	2- Full	Methylprednisolone Thymoglobulin Rituximab Tacrolimus MMF	Prednisone Tacrolimus MMF	(none identified)	Patient 1: Grade II POD 7, 441, 358, 269; Grade III POD 28, 31	Patient 1: Grade II POD 28, 402, 710 (POD 745) Patient 2 follow up POY 1	(43–45)
Antalya, Turkey	4- Full 1- Partial	Prednisone Thymoglobulin Tacrolimus	Prednisolone Tacrolimus MMF	Infection: Viral (pneumonia) patient 1 POM 24, Bacterial (infraorbital abscess) patient 3 POM 6, Fungal (pulmonary and cerebellar aspergillosis) patient 4 POM 9 Graft loss: Patient 4 graft removal POM 10 Death: Patient 4 passed away POM 11 (sepsis) Hematologic: Neutropenia patient 3 POM 3 Malignancy: Patient 4 squamous cell carcinoma POM 5 & post-transplant lymphoproliferative disorder POM 6	No	Patient 1: Grade II POM 12, 15, 20, 22, 30, 36, 40, 55; grade III POM 15 (POM 65) Patient 2: Grade II POM 24 (POM 60) Patient 3: Grade II POM 15 (POM 47) Patient 4: Grade II POM 10 (POM 11) Patient 5: Grade II POM 24 (POM 42)	(46)
Gliwice, Poland	2- Full	Methylprednisolone Thymoglobulin Tacrolimus MMF	Methylprednisolone Tacrolimus MMF	(none identified)	yes, but none detected	Patient 1: Grade II POD 34 (34 POM) Patient 2: Grade II POD 34 (19 POM)	(47, 48)
Barcelona, Spain	1- Full 1- Partial	Prednisone Thymoglobulin	Prednisone Tacrolimus MMF Sirolimus (replaced MMF due to rejection)	Other: Oro-cutaneous fistula patient 1 POD 17, Parotid sialocele patient 1 POD 28, venous thrombosis of the left external jugular and left retromandibular vein anastomoses patient 1 POD 3	Patient 1: Grade II/III POD 28	Patient 1: Grade II/III POD 28, grade II POD 75 (POM 4) Patient 2: Not reported	(49, 50)
Lyon, France (Amiens)	3- Partial	Prednisone Thymoglobulin Tacrolimus MMF Donor hematopoietic stem-cell transplant Serial extracorporeal photochemotherapy (1 patient)	Prednisone Tacrolimus MMF Patient 1: Sirolimus (added to Tacrolimus) and then both discontinued and replaced with Everolimus due to mild thrombotic microangio-pathy &increased creatinine, Sirolimus was replaced with Tacrolimus POM 116 due to chronic rejection Patient 2: Adding Sirolimus to reduce Tacrolimus	Infection: Viral (HSV1, EBV, Poxvirus)& Fungal (candida stomatitis) patient 2 POD 185 Renal: Elevated creatinine Patient 1 POM 83 Graft Loss: Patient 1 (POY 10) Death: Patient 1(small cell carcinoma POY 11) Hematologic: Thrombocytopenia, hemolytic anemia Malignancy: Patient 1 small cell carcinoma POY11 Patient 2 post-transplant monoclonal B-cell lymphoma POM 5 & hepatic EBV associated post-transplant smooth muscle tumors- POM 24 Other: Mouth ulcers patient 2 POM 33	Patient 1: Grade III POM 94 Patient 2: Grade II POD Grade III POD 41, 186	Patient 1: Episode (grade not specified) POD 18, 214, grade III POM 94, 102 (POY 11) Patient 2: Grade II POD 239, 474 Grade III 103, 186, 527, 541, 931 (POY 6) Patient 3: Not specified	(51–54)
Sevilla/Valencia, Spain	2- Partial	Methylprednisolone Basiliximab Tacrolimus	Prednisone Tacrolimus MMF Sirolimus (replaced Tacrolimus patient 1 due to Tumor)	Infection: Viral (CMV viremia) patient 2 POW 3&7, Bacterial (bacteremia, tracheobronchitis, surgical site infection) patient 2 through POD 47 Malignancy: Pseudosarcomatous spindle-cell tumor patient 1 POM 11	No	Patient 1: Grade III POD 14, 350 (POM 16) Patient 2: None detected (POW 70)	(55, 56)
Paris, France	5- Full (including 1 retransplant) 2- Partial	Methylprednisolone Thymoglobulin *For retransplant desensitization:* Methylprednisolone Thymoglobulin Rituximab Belimumab Plasma exchange IV Ig	Methylprednisolone Tacrolimus MMF Extra-corporeal photophoresis	Infection: Patient 1: CMV infection POM 2, CMV viremia POM 7 Patient 2: labial HSV-1 POM 1, cellulitis POY 4 Patient 3: pseudomonal infection POM 2 Patient 5: CMV viremia POM 3 Patient 6: Pneumonia POD 1 Patient 7: mandibular septic pseudarthrosis POM 3, CMV infection POM 3 Patient 8: S. aureus sepsis POY 8 after graft removal, norovirus POM 2, CMV oesophagitis, 2 episodes of aspiration pneumonia POM 3&8 Renal: Renal failure patient 1 POM 3, patient 5 POY 2, patient 6 POM 4&POY 1, patient 7 POM 3 Graft Loss: Patient 5 POY 8 (chronic rejection) Death: Patient 3 POD 65 (pseudomonal infection) Patient 7 POY 3.5 (suicide) Metabolic: Hypertension patient 1 POY 7, patient 2 POY 6&patient 5 POY 2, Hypercholesterolemia patient 2 POY 6, patient 4 POY 4, patient 5 POY 2, patient 6 POY 1, hypertriglyceridemia Hematologic: Thrombotic microangiopathy patient 5 POY 2 Other: Delirium patient 8, brief hypoxic cardiac arrest patient 8 POM 3&8, pseudoarthrosis patient 4 POM 1, depression patient 7 POY 1, venous thrombosis patient 4 POM 1	Patient 2: grade IV POY 1 Patient 5: grade III POY 3 Patient 8: Grade III POD 14	Patient 1: Grade II POM 1, 2, 3 & POY 5&6, grade III POY 3&10 (POY 9.5) Patient 2: No skin episodes (POY 7.1) Patient 3: No rejection detected (POD 65) Patient 4: Grade II POY 3, grade III POY 4&6, grade IV POY 6 (POY 6.7) Patient 5: Grade 2 POM 3, grade III POY4, chronic rejection led to graft removal POY 8 (POY 8) Patient 6: Grade II POM 3 (POY 5) Patient 7: None detected (POY 3.5) Patient 8: Grade III POD 14 (POM 30)	(57–60)
Saint Petersburg, Russia	1- Partial	Methylpred-nisolone Basiliximab MMF	Methylprednisolone MMF Cyclosporine A Tacrolimus (replaced cyclosporine A due to GvHD)	Hematologic: DIC, Anemia, Thrombocytopenia timepeoint not specified Other: Acute Respiratory Distress Syndrome, Systemic Inflammatory Response Syndrome timepoint not specified; donor vein thrombosis POD 1, Pseudaneurysm donor artery POD 52, GvHD POY 2	No	No episodes detected through POY 2	(61)
Xi’an, China	1- Partial	Methylprednisolone Anti-IL-2 mAb Tacrolimus MMF	Prednisone Tacrolimus MMF	Infection: Pneumonia (POM 1) Metabolic: Hyperglycemia POM 1, Diabetes mellitus POM 3 Death: due to non-adherence that led to chronic rejection, sepsis and organ failure	No	Acute rejection episodes at POM 3, 5, 17 (POY 2)	(62, 63)
Ghent, Belgium	1- Partial	Methylprednisolone Thymoglobulin Tacrolimus MMF	Methylprednisolone Tacrolimus MMF	Infection: Bacterial (sinusitis POW 17, pneumonia POD 40), Fungal (jaw abscess & pulmonary aspergilloma POM 11) Renal: Nephrotoxicity POM 11 Metabolic: Osteoporotic vertebral fractures POM 7 Other: Palato-fistula POM 1, SIADH POD 23 & 40	Grade IV POW 17	None detected (POM 24)	(64)
Rome, Italy	1- Partial	Methylprednisolone Thymoglobulin Tacrolimus MMF	N/A	Graft Loss: Facial allograft failure 2 days post-transplant (graft removed and replaced with latissimus dorsi-serratus anterior flap)	N/A	N/A	(65)
Rochester, USA	1- Partial	Thymoglobulin	Prednisone Tacrolimus MMF	Infection: Viral (CMV viremia) POM 6 Hematologic: Recurrent leukopenia POM 7	No	Not detected (POM 20)	(66)
Baltimore, USA	1-Full	Methylprednisolone Alemtuzumab	Steroid Tacrolimus MMF	(none identified)	No	Not specified (POM 10)	(67)

T-cell mediated rejection (TCMR) graded according to the Banff Classification 2007.

MMF, mycophenolate mofetil; AKI, acute kidney injury; GvHD, graft versus host disease; MPA, mycophenolic acid; CKD, chronic kidney disease; CMV, cytomegalovirus; DIC, disseminated intravascular coagulation; HSV, herpes simplex virus; VZV, varicella zoster virus; EBV, Epstein-Barr Virus; mAb, monoclonal antibody; IVIg, intravenous immunoglobulin; GI, gastrointestinal; POD, postoperative day; POW, postoperative week; postoperative month; POY, postoperative year.

**Table 2:** Immunosuppressive induction and maintenance regimens and significant outcomes reported across VCA hand transplantation centers.

Center	Number of patients	Immunosuppressive induction therapy utilized	Starting immunosuppressive maintenance therapy utilized	Significant outcomes reported (per patient/timepoint)	TCMR episodes (maximal follow up)	Reference
Lyon, France	2—Bilateral Hand 1—Bilateral Mid Forearm 1—Bilateral Palm 1—Bilateral Distal Forearm 1—Unilateral Hand	Prednisone Thymoglobulin Basiliximab Tacrolimus MPA MMF	Prednisone Tacrolimus MMF MPA Sirolimus (replaced Tacrolimus due to nephrotoxicity in patient 5)	Infection: HSV patient 5 POD 867, CMV infection patient 7, Osteitis left ulnar patient 2 POD 152, Cellulitis patient 5 POD 81 Kidney: nephrotoxicity patient 5 POM 26, increase creatinine patient 6 POM 1 &7 Graft Loss: chronic rejection following non-adherence patient 7 POD 4,680 Metabolic: Hyperglycemia patient 1 POD 30, 5 POD 10 and patient 6 POM 1, Osteopenia patient 1 and patient 4, osteoporosis patient 2, Diabetes mellitus patient 7 Other: Serum sickness patient 1 POD 1, thrombosis ulnar artery patient 2 POD1 & patient 5 POD 12	Patient 1:Grade II POD 53, 72 (POY 13) Patient 2: Grade II POD 57, 86, 27,59 (POY 10) Patient 4: Grade II POD 65 (POY 5) Patient 5: Grade III POD 10, 350, 560 (POY 4) Patient 6: Grade N/A rejection POD 57, 63 (POY 12) Patient 7: Grade N/A POD 76, 2,653, 4,400, 4,500 (POY 13)	(68–74)
Monza, (Italian Institute for Hand Surgery and Microsurgery) Italy	2—Unilateral Hand	Basiliximab MMF Tacrolimus Steroids	Tacrolimus Prednisone MMF	Infection: CMV & bacterial infection of allograft, not specified (patient 1) Kidney: creatinine increase (patient 1) Metabolic: Diabetes mellitus (patient 1)	Patient 1: Grade II POD 16, 271, 951, grade III POD 635, 1,365, 1,855 (POY 6) Patient 2: one episode grade III POM 27 (POY 5)	(75, 76)
Monza (S. Gerardo Hospital), Italy	2—Bilateral Hand	Basiliximab MMF Tacrolimus Steroids	Tacrolimus Prednisone MMF	Graft loss: one patient due to mismatch donor & recipients vessels (not specified which patient)	Patient 1: none reported (POY 1) Patient 2: none reported (POY 10)	(77, 78)
Louisville, USA	4—Unilateral Hand 1—Unilateral Forearm 1—Bilateral Hand	Methylprednisolone Thymoglobulin Basiliximab Alemtuzumab Autologous mesenchymal stem cell Transplant	Prednisone Tacrolimus MMF Sirolimus (replaced MMF to allow targeting of lower Tacrolimus levels in patient 2)	Infection: CMV infection patient 1 POM 3, CMV infection in patient 3 POM 2 Renal: Renal function decline patient 5 Metabolic: Post-transplantation diabetes mellitus patient 2 POM 2, weight gain patient 5 Malignancy: Marginal Zone Lymphoma patient 3 POY 2 Graft loss: patient 4 POM 9 due to unmanageable ischemia Other: Osteonecrosis both hips patient 2 POY 2&6, posttransplant lymphoproliferative disorder patient 3 POY 2	Patient 1: Three episodes within POY 1 (POY 12) Patient 2: Five episodes within POY 1, 5, 7 (POY 10) Patient 3: Three episodes through POY 2 (POY 4) Patient 4: Three episodes through POY 8 (POY 9) Patient 5: Five episodes grade II POY 1 (POY 2) Patient 6: No episodes detected (POM 6)	(79–81)
Innsbruck, Austria	2—Bilateral Hand 1—Unilateral Hand 2—Proximal Forearm & Hand	Methylprednisolone Alemtuzumab	Prednisone Tacrolimus MMF Sirolimus Everolimus 4 patient received Belatacept additionally due to kidney failure (patient 1, 2, 3, 5)	Infection: Viral (CMV infection in patient 1, CMV viremia in patient 2, 3, 4; HPV patient 4, HSV, VZV), Bacterial (C. diff) patient 1, Fungal (cutaneous), Scabies Renal: Increase in serum creatinine, hyperuricemia, renal failure with dialysis and kidney Tx POD 191 patient 2; increased creatinine patient 3 POY 3 Graft loss: 1 patient- 7 years post-transplant (chronic rejection) Metabolic: Hyperlipidemia patient 4, hypercholesterolemia type 2 diabetes mellitus patient 4, hyperglycemia, hypertension, osteopenia, serum sickness GI: Vomiting, Diarrhea Malignancy: Basal cell carcinoma, nasal keratoacanthoma, bullous pemphigoid Other: Dermatological (diffuse erythema), visual acuity loss, mental confusion, headache with high Tacrolimus level patient 1	Patient 1: Six episodes through POY 3, grade II POY 9 (POY 9) Patient 2: Six episodes between POD 50 and POY 6, grade II (POY 9) Patient 3: Two episodes POD 15 (POY 1.5) Patient 4: Grade II POD 55 Patient 5: Grade II POM 2	(76, 82–91)
Brussels, Belgium	1—Unilateral Hand	Thymoglobulin	Prednisone Tacrolimus MMF (temporarily discontinued due to diarrhea)	Diarrhea	Grade III POM 43 (POY 5)	(76, 92)
China	1—Bilateral Hand 5—Unilateral Hand 3—Unilateral Forearm 2- Bilateral Forearm 1-Palm 1-Thumb	Methylprednisolone Prednisone Thymoglobulin Tacrolimus MMF Cyclophosphamide	Prednisone Tacrolimus MMF	Infection: Viral (CMV infection patient 11 POD 70), Bacteria (TB patient 1 POM 6), Fungal (cutaneous infection) patient 1 POM 15&patient 2 POM 15, pulmonary infection patient 3, postoperative wound infection patient 4 Metabolic: Hyperglycemia patient 2, 5, 6, 7 & 9, Cushing syndrome, elevated transaminases, hypertension patient 5 POY 6, Hypoproteinemia patient 3&4 GI: Diarrhea patient 2 POM 1 Graft loss: patient 2 POY 2 due to ischemic changes; patient 3 POY 1 due to withdrawal of IS in the course of unmanageable pulmonary infection; patient 4 partial (thumb) POY 1 due to decrease of IS in the course of chronic wound healing disorder; chronic rejection in patient 8 led to graft loss POY 2; graft loss due to non-adherence in patient 10 POY 1; graft loss due to non-adherence in patient 11 POY 2; graft loss due to non-adherence POY 2 Other: Eczema patient 9, Dermatitis patient 1 POW 6 &patient 2 POW 7, intraoperative arterial thrombosis patient 1	Patient 1: Rejection every year of follow up (POY 10) Patient 2: None detected (POY 2) Patient 3: Once postoperatively (POY 1) Patient 4: One episode (POY 1) Patient 5: Every year (POY 9) Patient 6: Every year (POY 8) Patient 7: Every year (POY 7) Patient 8: Rejection episode POM 6, POY 2 (POY 2) Patient 9: Episode POY 1, 3, 5 & 6 (POY 6) Patient 10: Episode POM 7 (POY 1) Patient 11: Episode POW 8 (POY 2) Patient 12: Episode POY 2 (POY 2)	(93, 94)
Valencia, Spain	2—Bilateral Hand 1- Bilateral Trans-Humeral Arm	Methylprednisolone Alemtuzumab	Prednisone Tacrolimus MMF Sirolimus (replaced Tacrolimus due to increase creatinine)	Infection: Fungal (cutaneous infection) patient 2 Metabolic: Diabetes patient 1 POM 2, hypertriglyceridemia patient 2, hypertension patient 1 through POM 6 Renal: increased creatinine patient 1 POM 10, also patient 2&3 Hematological: Anemia patient 3 Other: Dermatological (dermatitis, mouth ulcers, hand rash) in patient 2, loss of visual acuity patient 1 POD 190	Patient 1: Grade III POM 6, 13 & 26 (POM 26) Patient 2: No episodes detected (POD 668) Patient 3: One episode POD 68 (POD 542)	(95, 96)
Milan, Italy	3—Unilateral Proximal Forearm 1- Bilateral Hand	Methylprednisolone Basiliximab Autologous mesenchymal stem cells	Prednisone Tacrolimus MMF	Infection: Viral (CMV viremia) in 2 patients, Bacterial (Intestinal Clostridium) in 1 patient Hematological: Mild Anemia (2 patients) Renal: increased creatinine in 1 patient Metabolic: Hyperglycemia (2 patients)	Patient 1: None reported (POM 29) Patient 2: None reported (POM 18) Patient 3: None reported (POM 7)	(97–99)
Trzebnica, Poland	4—Unilateral Hand 1—Unilateral Midforearm 1—Bilateral Hand	Methylprednisolone Basiliximab Tacrolimus MMF	Steroids Tacrolimus MMF	Infection: Viral (CMV infection patient 3, patient 4 POD 28 Herpes zoster), Bacterial (acute tonsilitis) Renal: CKD in 2 patients Graft loss: graft loss due to thrombosed arteries in graft in patient 3 POD 2, amputation of necrotis of distal phalanges due to thrombosed arteries in patient 5 POD 13 Metabolic: Hyperglycemia patient 1 through POY 2 and two more patients, hyperuricemia in 2 patients, dyslipidemia in 3 patients, diabetes mellitus in 2 patients, hypertension in 2 patients	Patient 1: None reported (POY 5) Patient 2: None reported (POY 4) Patient 3: None reported (POD 2) Patient 4: Grade II POW 6 (POY 3) Patient 5: Grade II POW12 (POY 2) Patient 6: None reported (POY 1)	(100–103)
Georgia, USA	1- Unilateral Distal Forearm	Thymoglobulin	Steroids Tacrolimus MMF Belatacept (replaced Tacrolimus/MMF due to increased creatinine) Sirolimus (replaced Tacrolimus/MMF)	Renal: Nephrotoxicity, proteinuria through POY 1	Grade II POD 90, 129 (POM 42)	(104, 105)
Mexico City, Mexico	1—Bilateral proximal forearm 1—Bilateral Arm 1—Bilateral total arm (R) and midarm (L)	Thymoglobulin	Prednisone Tacrolimus MMF	Infection: Viral (Herpes, sinusitis) patient 3 POM 12, Bacterial (pneumonia) Death: Patient 1 (suspected transfusion-related acute lung injury or cytokine Storm POD 1) Metabolic: Osteoporosis, Hyperglycemia patient 3 POM 2, Vitamin D deficiency Other: axonal motor neuropathy of the peroneal nerves	Patient 1: N/A Patient 2: Grade II POD 385, 522, 766 (POY 2) Patient 3: Grade II POD 18, 89, grade III POD 54 (POY 3)	(106–108)
North Carolina, USA	1—Unilateral Proximal Forearm	Thymoglobulin	Prednisone Belatacept Tacrolimus MMF Sirolimus (replaced Tacrolimus)	Infection: Viral (HSV) POY 1 Hematological: Neutropenia POM 6 Renal: increased creatinine POM 4 Other: Dermatological (mouth ulcers, skin lesions, erythema multiforme) POY 1, Neurological (tremor) POM 4, thrombosis in graft artery POD 1	Grade III POM 8 (POM 20)	(109)
Kaohsiung, Taiwan	1—Unilateral Hand 1- Unilateral Forearm	Methylprednisolone Thymoglobulin	Prednisone Tacrolimus MMF	Infection: Pneumonia patient 1 POD 119 Hematological: Leukopenia patient 1 POD 119 Other: Avascular necrosis of both hip joints patient 1 POY 2.5	Patient 1: Episodes at POD 105, 810 (POY 4) Patient 2: Grade II POD 63 (POY 2)	(110–112)
Philadelphia, USA	1—Bilateral Hand 1—Pediatric Bilateral Hand	Thymoglobulin	Prednisone Tacrolimus MMF	GI: Diarrhea Renal: decreased renal function patient 2 POM 7 Hematological: Anemia	Patient 1: None reported (POY 5) Patient 2: Grade II POD 8 (POY 1)	(113, 114)
Leeds, UK	4—Bilateral Hand 2—Unilateral Hand	Prednisone Alemtuzumab	Prednisone Tacrolimus MMF	(none identified)	Patient 1: Grade II POD 97, 163, 888, 917, Grade III POD 51, 149, 198, 756, 1,153, 1,317, 2,256 (POY 7) Patient 2: Grade II POD 59, 146, 198, 230 (POY 3) Patient 3: Grade II POD 75, 678 (POY 2) Patient 4: Grade II POD 57, 92, 127, 191, Grade III POD 136 (POY 1) Patient 5: Grade II POD 122, 186 (POY 1) Patient 6: None detected (POM 10)	(115)
Melbourne, Australia	1—Unilateral Hand	Basiliximab	Prednisolone Tacrolimus MPA	Metabolic: Hyperglycemia POD 7, hypercholesterolemia POY 2	Grade II POD 10 (POY 2)	(116)
Baltimore, USA	1—Bilateral Forearm 2—Unilateral Arm	Bone Marrow Cell Infusion	Prednisone Tacrolimus	Other: Rheumatoid Arthritis patient 1 POY 3	Patient 1: None reported (POY 3)	(117, 118)
Brigham and Women's, Boston, USA	1—Bilateral Upper Extremity 1—Bilateral Mid Forearm	Thymoglobulin	Prednisone Tacrolimus MMF	Infection: Bacterial (pneumonia patient 1, femoral catheter bacteremia) Other: Appendicitis patient 1	Patient 1: Two episodes grade III through POY 2 (N/A) Patient 2 2014: Grade II POY 2 (POY 4)	(32, 119–121)
Massachusetts General Hospital, Boston, USA	1—Unilateral Hand	Methylprednisolone Thymoglobulin MMF	Prednisone Tacrolimus MMF	(none identified)	None reported (POY 1)	(122)
Cochin, India	2—Bilateral Hand	Thymoglobulin	Prednisolone Tacrolimus MPA	Infection: Viral (herpes labialis patient 1 POM 6, upper respiratory tract patient 1 POM 8), Paronychia patient 1 Metabolic: Hypertension patient 1 POM 2 GI: Diarrhea patient 1 POM 2, patient 2 POM 18 with 12 kg weight loss	Patient 1: Grade II POM 4 & 9, grade III POM 8 (N/A) Patient 2: Grade III POM 1 (N/A)	(123)
Amrita, India	1—Bilateral Upper Arm (supracondylar) 1—Bilateral Proximal Forearm	Methylprednisolone Thymoglobulin Tacrolimus MMF	Prednisolone Tacrolimus MMF	(none identified)	None reported	(124, 125)
Pittsburgh, United States	1—Unilateral Hand 2—Bilateral Hand 2—Bilateral Forearm	Methylprednisolone Alemtuzumab	Tacrolimus Monotherapy plus single Posttransplant donor BM cell infusion POD 14	Renal: increased creatinine patient 5 Metabolic: Hyperuricemia patient 1 Other: deep vein thrombosis patient 3	Patient 1: Grade II POD 43, POM 21, grade III POM 13 (POY 3) Patient 2: Grade III POD 270 (POY 3) Patient 3: Grade II POD 25, 66, grade III 43 (POY 2) Patient 4: Grade II POD 18 (POY 2) Patient 5: Grade III POD 51 (POY 2)	(126, 127)
San Antonio, United States	1—Unilateral Hand	Thymoglobulin	Prednisone Tacrolimus MMF	Infection: Viral (CMV viremia) twice Renal: Two episodes of acute renal failure	Four episodes were reported (POM 9)	(128)
Pondicherry, India	1—Bilateral Forearm 1—Bilateral Trans-Humeral Arm	Thymoglobulin	Prednisolone Tacrolimus MMF	Renal: Myoglobinuria patient 1 POW 1 Metabolic: Diabetes patient 2 POW 2	Patient 1: Non reported (POY 2) Patient 2: Episodes POW 2, 12 (POY 2)	(129, 130)
Seoul, South Korea	1—Unilateral Distal Forearm	Steroids Basiliximab Tacrolimus	Steroids Tacrolimus MMF	Neutropenia POD 44	Grade II POD 33, grade III POD 41	(131)
Antalya, Turkey	1—Bilateral Proximal and Distal Forearm 1—Bilateral Middle and Proximal Forearm	Prednisolone Thymoglobulin	Prednisolone Tacrolimus MMF	Death: 1 patient (heart/kidney failure, POD 100) Metabolic: Hyperglycemia	None reported	(132, 133)
Nijmegen, Netherlands	1—Bilateral Proximal Forearm	Thymoglobulin	Prednisone Tacrolimus MMF	Other: Mild tremor, hair loss, loss of appetite	None reported (POY 1)	(134)
Los Angeles, USA	1—Unilateral Proximal Forearm	(none specified)	Prednisone Tacrolimus MMF	(none specified)	Grade II POD 463 (POD 475)	(135, 136 )

T-cell mediated rejection (TCMR) graded according to the Banff Classification 2007.

MMF, mycophenolate mofetil; MPA, mycophenolic acid; HSV, herpes simplex virus; UTI, urinary tract infection; CMV, cytomegalovirus; HPV, human papillomavirus; VZV, varicella zoster virus; TB, tuberculosis; CKD, chronic kidney disease; POD, postoperative day; POW, postoperative week; postoperative month; POY,postoperative year.

**Table 3:** Immunosuppressive induction and maintenance regimens and significant outcomes reported across VCA centers that performed simultaneous face and hand transplantation.

Center	Number of patients	Induction	Maintenance	Significant immunosuppressant outcomes (per patient/timepoint)	Mucosa used as surveillance tool (y/n)	TCMR episodes (maximal follow up)	Reference
New York, USA	1- Full Face, Bilateral Hands	Methylprednisolone Thymoglobulin Rituximab	Prednisone Tacrolimus MMF	(none identified)	No	None (until 2022)	(137)
Boston/New Haven, USA	1- Full Face, Bilateral Hands	Methylprednisolone Thymoglobulin Tacrolimus MMF	Prednisone Tacrolimus MMF	Infection: Viral (norovirus gastroenteritis), Bacterial (septic shock, C. diff diarrhea, conjunctivitis, zygomatic fluid collection) POD 2, Pneumonia (POD 1), limited renal insufficiency (until POD 17) Graft Loss: Right and Left Hand loss- day 5 post-transplant (irreversible ischemia from septic shock) Hematologic: Leukopenia POD 2	No	None (until POD 38)	(138)
Paris, France	1- Full Face, Bilateral Hands	Methylprednisolone Thymoglobulin Tacrolimus MMF	Prednisone Tacrolimus MMF	Infection: Bacterial (Pseudomonal infection of all allografts); Shock POD 3; Pneumonia POD 3 until POD 17 renal insufficiency POD 3 until POD 17 Graft Loss: Left hand allograft and upper third of facial allograft POD 5 Death: Patient 1Anoxic cardiac arrest (day 65 post-transplant)	No	1 episode (Grade I) at POD 3 until POD 35	(138)

T-cell mediated rejection (TCMR) graded according to the Banff Classification 2007.

MMF, mycophenolate mofetil; POD, postoperative day.

### Face transplantation

**Figure 3:**
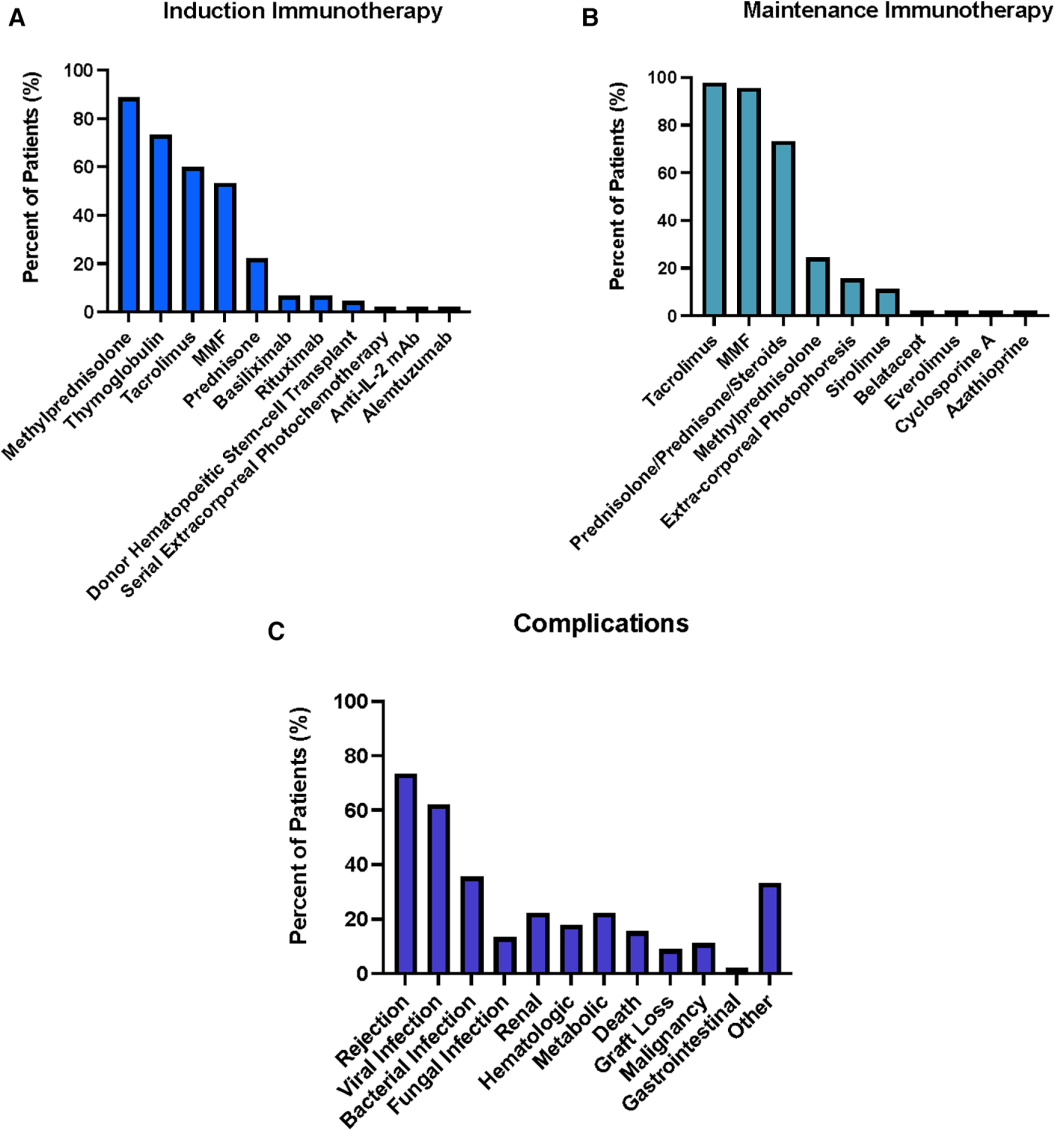
Summary of Induction (**A**) and Maintenance (**B**) Immunosuppressive Therapies used for 45 face transplant patients across 18 centers. Percentages represent percent of total patients treated with given immunosuppressive agent. Summary of complications (**C**) associated with the post-transplantation period for face transplants across international centers. Renal includes nephrotoxicity, increased creatinine levels, renal abscess, dysfunction, urinary tract infection, sepsis, acute kidney injury; Hematologic includes Neutropenia, Leucopenia, Thrombocytopenia, hemolytic anemia, thrombocyte microangiopathy; metabolic includes weight loss, osteoporosis, hypertension, hyperglycemia, diabetes mellitus, hypertriglyceridemia, hypercholesterinemia; gastrointestinal (GI) includes diarrhea, ulcer; other includes Delirium, brief hypoxic cardiac arrest, pseudoarthrosis patient, depression, venous thrombosis patient, Acute Respiratory Distress Syndrome, Systemic Inflammatory Response Syndrome, donor vein thrombosis, Pseudoaneurysm donor artery, fistula.

A total of 18 face transplant centers were identified. Among the evaluated induction therapies as shown in Figure 3A, Thymoglobulin exhibited the highest utilization rate, being employed in 40/45 patients (88.9%). Methylprednisolone was the second most common induction immunosuppressive utilized in 33/45 (73.3%) patients. MMF, Tacrolimus, and prednisone were the next most commonly utilized agents utilized in 27/45 (60.0%), 24/45 (53.3%), and 10/45 (22.2%) patients. Other induction agents utilized in a small minority of patients included basiliximab (3/45; 6.7%), donor hematopoetic stem-cell transplant (3/45; 6.7%), rituximab (2/45; 4.4%), extracorporeal photochemotherapy (1/45; 2.2%), anti-IL-2 mAb (1/45; 2.2%), and alemtuzumab (1/45; 2.2%). A table outlining the usage of less commonly utilized agents is presented in Supplementary Figure S2. In terms of maintenance therapies as shown in Figure 3B, Tacrolimus exhibited the highest adoption rate by transplant centers, utilized in 44/45 (97.8%) patients, followed by MMF (43/45; 95.6%), prednisolone/prednisone/steroids (33/45; 73.3%), and methylprednisolone (11/45; 24.4%). Other maintenance immunosuppressive therapies that were utilized in small proportion of patients by centers included extra-corporeal photophoresis (7/45; 15.6%), sirolimus (5/45; 11.1%), belatacept (1/45; 2.2%), everolimus (1/45; 2.2%), cyclosporine A (1/45; 2.2%), azathioprine (1/45; 2.2%) (Supplementary Table S1).

Among the observed complications, rejection was the most prevalent according to Figure 3C and was identified in 33/45 patients (73.3%). In detail: a total of 111 (100%) episodes of rejections were graded according to the Banff classification, additional 16 were reported without grading according to Banff. Out of all Banff graded rejection episodes, 48 episodes (43%) were grade II and 62 episodes (56%) were grade III while 1 episode (1%) was grade IV. Out of the Banff classification-graded biopsies, 25 (23%) episodes with grade II and 23 (21%) episodes grade III were seen within the first 12 months posttransplant; 12 (11%) episodes grade II and 9 (9%) episodes grade III within first 24 months; 4 (4%) episodes grade II and 5 (5%) episodes grade III in POY 3; 5 (5%) episodes grade II and 9 episodes grade III in POY 4; 1 (1%) episode grade II and 8 (8%) episodes grade III in POY 5; 1 (1%) episodes grade II, 5 (55) episodes grade III and 1 (1%) episode grade IV in POY 6; 2 (2%) episodes grade III POY 7 and 1 (1%) episode grade III POY 10. Episodes that were reported without Banff grading occurred as follows: 7 episodes within the first 12 months, 3 episodes in POY 2, 2 episodes in each POY 3 and 4, 1 episode in each POY 6 and 9. In summary, 48 (43%) of the Banff-graded biopsies occurred in the first 12 months posttransplant, 21 (19%) episodes in the second year, 9 (8%) episodes in the third year, 14 (13%) episodes in the fourth year, 9 episodes (8%) in the fifth year, 7 episodes (7%) in the sixth year, 2 (2%) in the seventh and 1 (1%) episode in the tenth year posttransplant. The next most common category of complications was infections, which was reported in 28/45 patients (62.2%). Within this category, viral infections were identified in 19/45 patients (42.2%). CMV was the most common subtype (11/45 patients; 24.5%) with CMV infections reported in 7 (15.5%) patients while CMV viremia was observed in 4 (8.8%) patients. Viral infections were followed by bacterial infections (reported in 16/45 patients; 35.6%) and fungal infections (reported in 6/45 patients, 13.3%). Renal and metabolic complications followed the infection category and were each reported in 10/45 patients (22.2%) with elevated creatinine (3/45; 6.7%), hypertension (4/45; 8.9%), and hypercholesterolemia (4/45; 8.9%) being the subcategories observed in most patients. Hematologic complications were reported in 8/45 patients (17.8%), with neutropenia/leukopenia dominating this category (reported in 5/45 patients, 11.1%). Deaths of face transplant patients were reported in a total of seven patients with three deaths occurring from systemic infections, two deaths from malignancies, one death from suicide, and one not specified. Graft loss was reported in 4/45 patients (8.9%) after a median follow up of 77.5 posttransplant months (range 10–120 months) while malignancy was reported in 5/45 patients (11.1%). Gastrointestinal (GI) complications were the least frequent complication reported, with diarrhea identified in 1/45 patient (5.6%). Other complications were identified in 15/45 patients with the most common subtype being fistula formation (5/45; 11.1%).

### Hand transplantation

**Figure 4:**
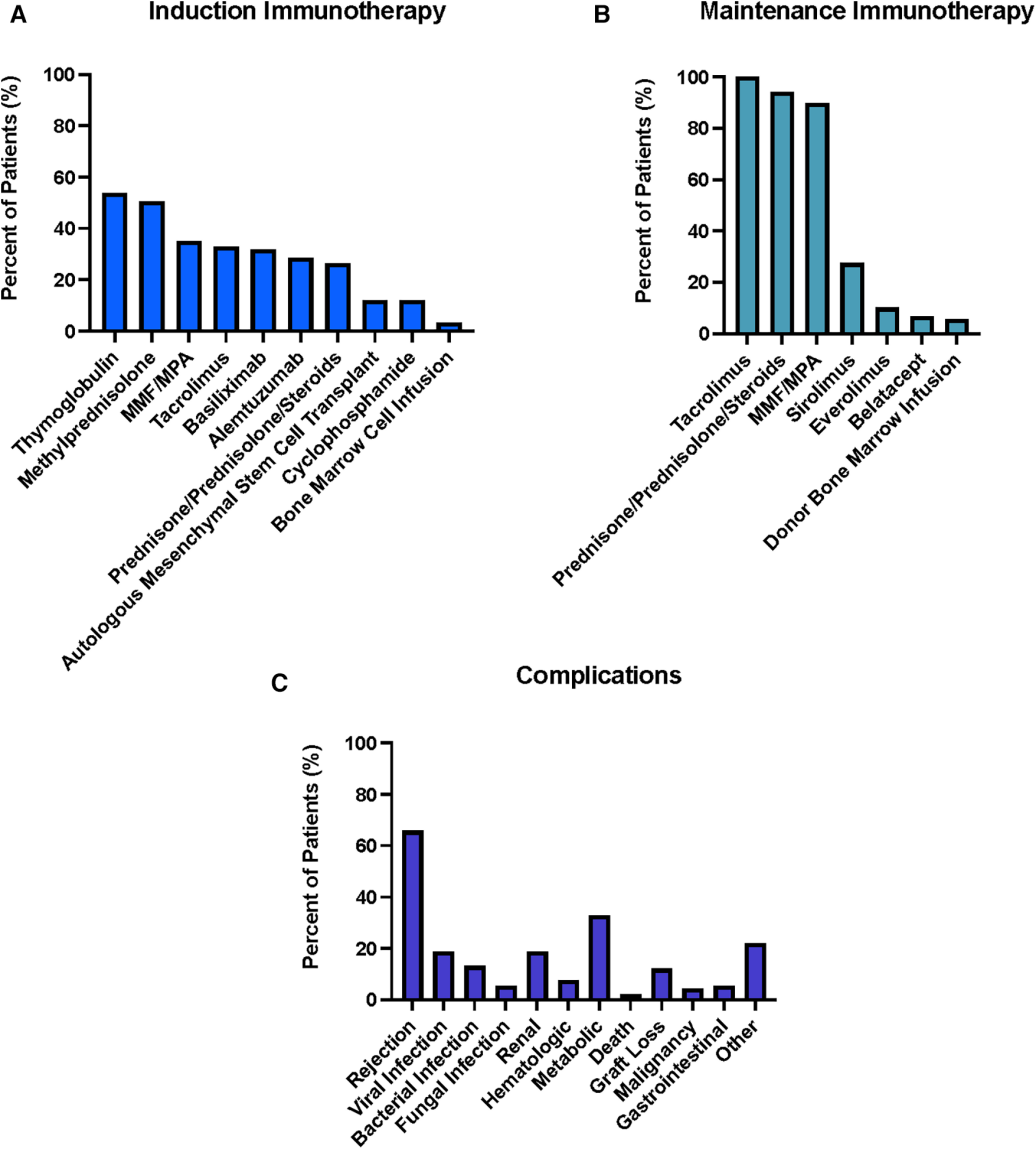
Summary of Induction (**A**) and Maintenance (**B**) Immunosuppressive Therapies used for 91 hand transplant patients across 29 centers. Percentages represent percent of total patients treated with given immunosuppressive agent. Summary of complications associated with the post-transplantation period for hand transplants across international centers (**C**). Renal includes nephrotoxicity, increased creatinine levels, kidney failure requiring kidney transplantation, urinary tract infection, sepsis, acute kidney injury, hyperuricemia, myoglobinuria, proteinuria; Hematologic includes Neutropenia, Leucopenia, anemia, proteinemia; Metabolic includes weight loss, osteoporosis, osteopenia, hypertension, hyperglycemia, diabetes mellitus, hypertriglyceridemia, hypercholesterinemia, hyperlipidemia, serum sickness, weight gain, Cushing syndrome; Gastrointestinal (GI) includes diarrhea, vomiting; Other includes donor vein thrombosis, serum sickness, osteonecrosis hip, posttransplant proliferative disorder, mental confusion, vision loss, erythema, arterial thrombosis, eczema, neuropathy, tremor, appendicitis, arthritis, deep vein thrombosis.

The induction and maintenance therapies utilized in this study demonstrated varying degrees of adoption among the 29 centers identified from the search results. The distribution of these induction and maintenance immunosuppressive therapies is summarized in Figure 4A,B. Among the induction therapies evaluated, Thymoglobulin exhibited the highest utilization, being employed in 49/91 patients (53.8%). Methylprednisolone was the second most used induction therapy utilized in 46/91 patients (50.5%), followed by MMF/MPA (32/91; 35.2%), Tacrolimus (30/91; 33.0%), Basiliximab (29/91; 31.9%), Alemtuzumab (26/91; 28.6%), and Prednisone/Prednisolone/Steroids (24/91; 26.4%). In a smaller proportion of patients autologous mesenchymal stem cell transplant (11/91; 12.1%), cyclophosphamide (11/91; 12.1%), and bone marrow cell infusion (3/91; 3.3%) were utilized as induction therapies. The most prevalent maintenance therapy observed in the study was Tacrolimus which was employed in all centers across all patients (91/91, 100%). Prednisone/Prednisolone/Steroids and MMF/MPA were the second most common agent classes utilized, identified in 86/91 (94.5%) and 82/91 (90.1%) of patients respectively. A smaller proportion of patients were treated with maintenance immunosuppressive medication utilizing sirolimus (25/91; 27.5%), everolimus (9/91; 9.9%), belatacept (6/91; 6.6%), donor bone marrow infusion (5/91; 5.5%).

The distribution of complications reported by various hand VCA centers is summarized in Figure 4C. Similar to face transplantation, the most prevalent complication was rejection as well, identified in 60/91 patients (65.9%). In total 158 episodes of rejection were reported, 74 episodes without a grading while 84 episodes were reported according to the Banff classification. Out of the 84 (100%) graded rejection episodes, 39 episodes (46%) were grade II while 16 episodes (20%) were grade III within the first 12 months posttransplant; 9 episodes (11%) were grade II and 4 episodes (5%) were grade III POY 2; 5 episodes (6%) were grade II while 7 episodes (9%) were grade III POY 3; 1 episode (1%) grade III was reported for each POY 4, 5 and 6. For the non-graded episodes, 30 episodes were reported within POM 12, 8 episodes in POY 2, 7 episodes in POY 3, 4 episodes in POY 4, 6 episodes in POY 5, 5 episodes in POY 6, 6 episodes in POY 7, 3 episodes in POY 8, 2 episodes in POY 9, 1 episode in POY 10 and 2 episodes in POY 12. In summary, out of the Banff graded rejection episodes 55 episodes (66%) occurred in the first 12 months posttransplant, 13 episodes (16%) in POY 2, 12 episodes (15%) in POY 3, 1 episode (1%) in each POY 4, 5 and 6. This is followed by infections, reported in 33/91 patients (36.3%). Within the category of infection, viral infections were the most common in 18/91 patients (19.8%) with CMV being the most common subtype in 12/91 patients (13.2%). Out of the 12 reported CMV events, 7 (7.7%) patients showed clinical signs of CMV infection while a CMV viremia was detected in 5 (5.5%) patients. Additionally, bacterial and fungal infections were identified in 12/91 (13.2%) and 5/91 patients (5.5%), respectively. This was followed by the category of metabolic complications, which was reported in 30/91 patients (33.0%) with the most common entity in this category being hyperglycemia identified in 22/91 (24.2%) patients. Renal complications were identified in 17/91 patients (18.7%) followed by hematological complications in 7/91 patients (7.7%). The most common hematological complication identified was anemia (4/91patients, 4.4%). GI complications were seen in 5/91 patients (5.5%) with diarrhea being the most common subcategorization seen in all those patients. Across all patients two resulted in death (2.2%) and 11/91 resulted in graft loss (12.1%). Graft loss was observed after a median follow up of 37.8 posttransplant months (range 9–152 months). Other complications were found in 20/91 patients with the most significant subtype being skin lesions/dermatitis in 6/91 patients (6.6%).

### Combined face and hand transplantation

**Figure 5:**
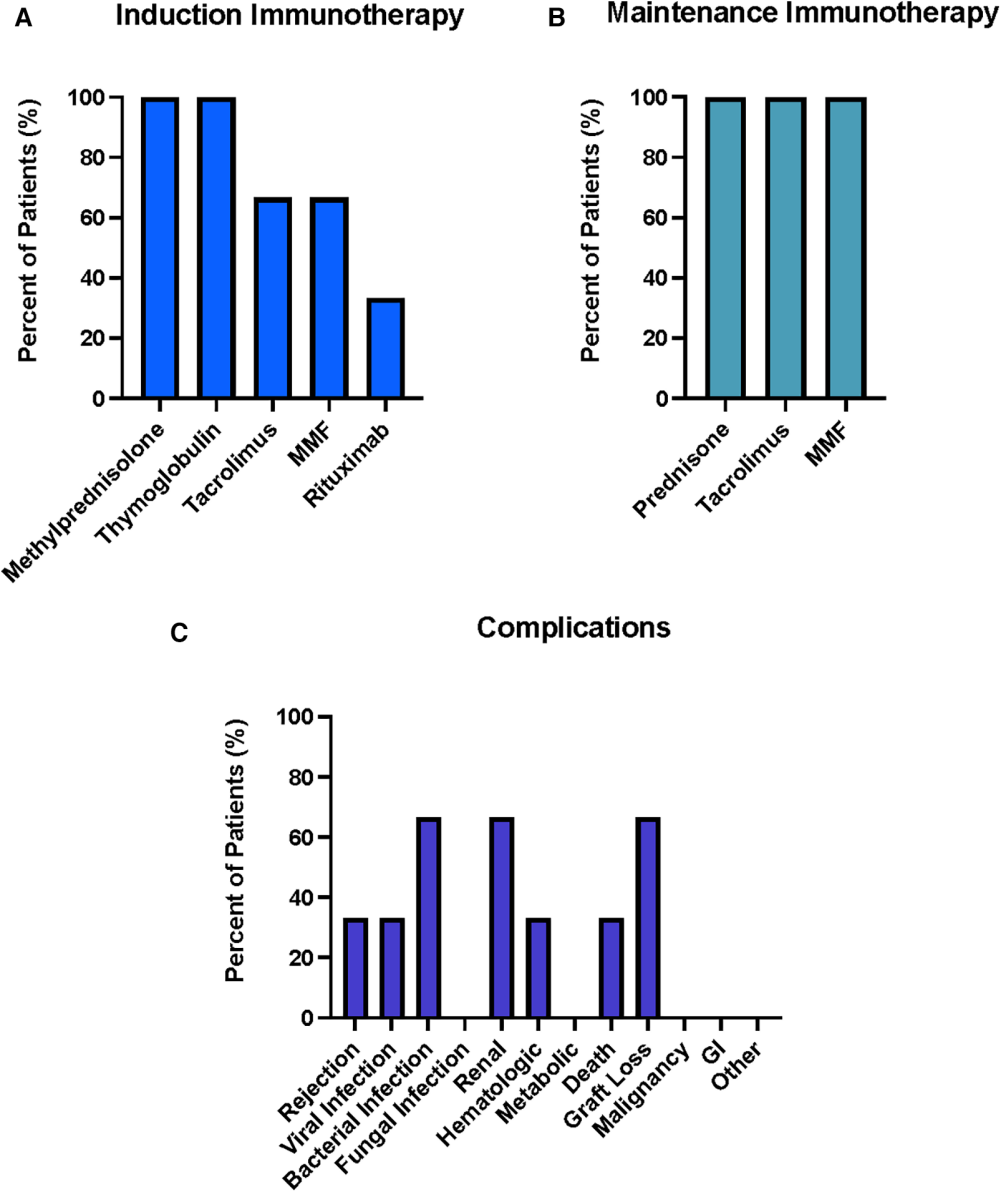
Summary of Induction (**A**) and Maintenance (**B**) Immunosuppressive Therapies used for three combined face and hand transplant patients across three different centers. Percentages represent percent of total patients treated with given immunosuppressive agent. Summary of complications associated with the post-transplantation period for face/hand transplants across three international centers (**C**). Complications were reported in detail with kidney insufficiency (renal) and leucopenia (hematologic).

Among the evaluated induction therapies as shown in Figure 5A, Thymoglobulin and Methylprednisolone were both utilized in all three patients (100%). Tacrolimus and MMF each exhibited utilization in 2/3 patients (66.7%) and rituximab was employed in 1/3 (33.3%) of patients to induce mature B-lymphocytes depletion. In terms of maintenance therapies, Prednisone, Tacrolimus, and MMF were each employed in all three patients (100%) as triple immunosuppressive therapy (Figure 5B).

As summarized by Figure 5C, both infections and renal complications were each reported in 2/3 patients (66.7%). Graft loss was a substantial concern, also reported in 2/3 patients (66.7%) within the first five postoperative days, indicating potential challenges in graft survival and function. Death, graft rejection, and hematologic complications were all each identified in 1/3 patient (33.3%). One episode of rejection was reported for one patient (33.3%) at POD 3.

## Discussion

The therapeutic paradigm and approach utilized in immunosuppression of VCAs has largely been adopted from solid organ transplant models and is outlined in Figure 2. A two-tiered approach is utilized, starting with an induction phase predominantly characterized by anti-lymphocyte mono-/polyclonal antibodies administered at high doses shortly before or at the time of transplant (18, 139). The goal of this phase is to achieve rapid and significant reductions in levels of T-cell lymphocytes soon after transplantation when risk of acute rejection is highest. Following this, a life-long maintenance phase is established utilizing a variety of agents designed to reduce T-cell functionality and activation including calcineurin inhibitors, mTOR inhibitors, and purine synthesis inhibitors (18, 19). Across both phases, steroid agents are utilized for additional immunosuppressive support. Data from SOT has demonstrated that implementation of immunosuppressive regimens has significantly improved long-term outcomes over the past three decades- offering an overall safe long-term therapy for improving allograft survival (140–142).

### Face transplantation

Our results in comparing immunosuppressant regimens across all identified face transplant centers revealed that thymoglobulin and methylprednisolone were the two most common agents utilized for induction, being utilized in the vast majority of patients (>70%). Thymoglobulin first became available over 30 years ago and prior to its adaptation to VCA, has been the most widely used lymphocyte-depleting preparation in solid organ transplantation (143). Its more prominent use in VCA over other lymphocyte depleting agents such as alemtuzumab or basiliximab is largely supported by previous kidney transplantation studies highlighting the superiority of thymoglobulin across several outcomes including reduced acute rejection, graft failure, and patient death (144–146). Methylprednisolone is often utilized as the steroid of choice during the induction phase due to its capacity to be given intravenously with ease intra- or peri-operatively as well as first line bolus therapy during episodes of acute rejection. In terms of maintenance immunosuppressive therapy, Tacrolimus, MMF, and steroids were by far the three most common agents utilized. The adaptation of this “triple therapy” approach in VCA has also been drawn from SOT that implemented this maintenance model which demonstrated increased survival rate of transplants with lower toxic side effects (147, 148). As it can be seen in Figure 3A where Tacrolimus and MMF were reported the third most common agents for induction, many centers often begin the long-term maintenance therapy at the peri-operative period (starting on day of surgery) in which the induction immunosuppressants are also given.

The most common complication category reported by centers in fVCA patients after rejection was infectious, reported in over 60% of patients. The most common infection type was viral with CMV being the most prevalent subcategory. This finding mirrors that seen in SOT where CMV is noted to affect up to three-quarters of all solid organ transplant recipients (149). An international multicenter study of CMV complications in fVCA patients by Kauke-Navarro et al. identified that patients with Donor + /Recipient—CMV allotransplantation status were at increased risk of CMV-related complications and that CMV infections were most likely to occur within the first-year after transplant when antiviral prophylaxis is discontinued (16). Given its high reported prevalence across the majority of fVCA patients with 26.7%, this highlights the importance of maintaining active surveillance for both the CMV seropositivity status of donors and recipients as well as signs of active infection following face transplantation in recipients. Slightly fewer events of CMV were reported for hand transplant patients with 19.3% while CMV has not been reported in patients receiving hand and face transplantation. Theoretically, CMV is present in the mucosa of face transplants and thus the rate of transfer, and clinically relevant infection may be higher in face as demonstrated here. Reported in approximately ∼20% of centers each, the next three most common complications were renal (renal failure, increased creatinine), metabolic (hypertension, diabetes/hyperglycemia), and hematologic (leukopenia); all of which have been identified as predominant side effects across the reciprocal immunosuppression agents utilized in both SOT and VCA models (150–153).

### Hand transplantation

The immunosuppressant regimens utilized for hand VCAs closely mirror those which were identified in fVCAs across different centers. The most common induction immunosuppressants used in over 50% of patients centers each was also thymoglobulin followed in conjunction with Methylprednisolone. Additionally, like face VCA, maintenance immunosuppression with the triple therapy regimen (Tacrolimus, MMF, and prednisone) was also the most common agents utilized in ∼90% of all hand VCA patients—a finding also corroborated in a separate review (154). This noted immunosuppression commonality between hand and face VCA is corroborated by a comparative study by Rifkin et al. which looked at 57 VCA and 98 kidney transplant patients and noted that hand and face VCA recipients received comparable MMF/prednisone doses and were treated with similar tacrolimus target trough levels as kidney recipients (139).

Additionally, the most common complications reported from hand VCA centers were also closely aligned with those of face VCA centers, which included infections, metabolic, and renal complications. This finding is largely congruent with the previous trends highlighted with face VCAs considering the most common immunosuppressant agents identified were the same across both face and hand VCA centers- thus a similar overall complication profile between hand and face may be expected and has been reported in similar studies (154). It's important to note that, CMV was once again the most common infection subtype reported in hand VCA centers, which further emphasizes the significance of having active CMV surveillance protocols for any type of transplant patient with VCA or SOT (155).

### Hand and face transplantation

To touch on briefly, our systematic review identified three cases across three different centers were a patient received both face and hand VCAs simultaneously as outlined in Table 3. All centers utilized thymoglobulin for induction and the standard triple therapy for maintenance. Interestingly, 2/3 patients experienced loss of their graft shortly after following the operation due to infectious complications. One patient lost their bilateral hand VCAs and one patient lost their left hand VCA and part of their face VCA before passing away from anoxic cardiac arrest about two months post-transplantation. Several factors have been considered for the relative increased percentage of graft failure in this patient population including increased antigenic burden, extended anesthesia time, and large-volume resuscitations that were required. However the patient population size is far too limited for any definitive conclusions to stand (138).

Overall, our results highlight that both face and hand VCA utilize similar immunosuppression protocols for induction (thymoglobulin & methylprednisolone) and maintenance therapy (Tacrolimus, MMF, Prednisone). However, modifications to the standard triple therapy are typically considered when a patient experiences intolerance to the triple therapy, such as a decline in renal function or the development of severe, refractory diarrhea. In such cases, as depicted in Figures 3, 4, the dose of tacrolimus may be reduced while belatacept may be added, or tacrolimus can be completely discontinued and replaced with, for example, sirolimus or everolimus. It is hypothesized that due to its mechanism of action as a T-cell costimulation blocker, belatacept could also potentially reduce the incidence of AMR (antibody-mediated rejection) but may be in an inferior position preventing acute cellular rejection episodes as shown by BENEFIT study in kidney transplant patients.

Especially if patients exhibit an uneventful course, it may also be contemplated to discontinue prednisone entirely and transition to a dual therapy, consisting of, for instance, Tacrolimus and MMF. The treatment is usually adjusted to the individual's specific situation due to the absence of guidelines for low case numbers and the patient's unique characteristics and can include a number of agents highlighted in Supplementary Figure S2.

Face and hand VCA differ through the incorporation of mucosal tissue in face VCA which studies have indicated might be more immunogenic than skin alone and may reject at a higher frequency (11, 12) and indeed our results revealed that rejection episodes and clinical CMV infections were seen more often in face than in hand transplant patients indicating that the complex fVCA composition could add to immunogenicity. However, centers reported a similar distribution of the remaining most frequent patient encountered complications. That being said, it is unknown if certain immunosuppressant agents or different dosages of established immunosuppressive regimens might have better indications for mucosal-sourced VCAs such as face. However, given the small global subset continued expansion of the current VCA patient population will need to occur in order to successfully evaluate and identify such potential trends. The current challenges still revolve around the long-term toxic side effects of immunosuppressants and the occurrence of rejection reactions during the course of treatment. An ideal therapy would, therefore, shift from the approach of immunosuppression to the approach of inducing tolerance. And, thus, to make VCA more accessible to a greater number of patients, especially those for whom a cost-benefit analysis currently yields negative results.

### The relative antigenicity of VCA tissues

In the field of VCA, it is widely accepted that skin is the most antigenic tissue (10, 156). As a result, clinical decisions in VCA management often rely on skin biopsy results, which are assessed using the Banff scale established in 2007, in conjunction with clinical evaluations of the graft (157).

The basis for this widely held belief and clinical practice comes from animal studies conducted with allogeneic split-thickness skin grafts in comparison to solid organ transplants in various animal models, including dog and rat (e.g., Moseley et al. 1966) (10). These studies globally indicated that skin was highly allogeneic, although some studies, like the one conducted by Lee et al., lacked conclusive evidence that skin was the most antigenic tissue in composite grafts (158, 159). Another study by Oda et al. assessed relative antigenicity in a rat hindlimb model and found that microRNA-155 expression, a marker of inflammation, was significantly higher in skin compared to bone (160). This led to the conclusion that skin was more antigenic during acute rejection.

However, it is important to note that none of these studies included models that incorporated mucosal tissue. In the context of limb VCA, skin is undeniably crucial due to its large surface area. However, in facial VCA, the addition of mucosal tissue becomes a significant consideration (11, 12).

In clinical practice, we have observed that oral mucosa in facial VCAs experiences a higher rate of rejection and more frequent rejection episodes (11–13, 161). This observations supports that mucosa is more antigenic than skin in facial VCAs. Surprisingly, there is a notable absence of studies investigating the comparative antigenicity of oral mucosa in comparison to skin. Moreover, there is a pressing need for additional research to unravel the mechanisms underlying both skin and mucosal rejection, ideally through direct comparison studies.

### Limitations

Although the search criteria of our systematic review were thorough in including the large majority of face and hand VCA cases reported, not all individual cases to date were able to be identified in the literature and included in this review. Furthermore, due to nature of this review analyzing published data and not conducting a multi-center study, we did not have access to updated data directly by the centers meaning this review is unable to capture all treatment changes or complications that occurred in the patients until today. Therefore, the maximal follow up period is listed in Tables 1–3 for each patient that has been reported.

## Conclusion

Although VCAs have the incredible ability to restore optimal functional and aesthetic outcomes to patients who are not candidates for other reconstructive options, they necessitate life-long adherence to immunosuppressive regimens. This review provides a contemporary update and comparison on the current immunosuppressive regimens utilized in face and hand VCAs around the world and ultimately identified that although there is notable variation between induction and maintenance immunosuppressive agents that are utilized, the most common approach across *both* hand and face VCA centers primarily includes utilization of thymoglobulin /methylprednisolone induction regimen with a tacrolimus/MMF/steroid maintenance regimen. Given the increased immunogenicity of VCA tissue in general and differences within VCA types such as hand compared to face that is including mucosa whose immunogenic role has not yet been fully investigate, relative to SOT, it is important that we continue to explore and develop immunosuppressive agents tailored to VCA graft types that work to provide maximal allograft health outcomes while minimizing the associated complications. Notably, facial grafts exhibited a heightened susceptibility to rejection episodes, higher grades of rejection, and clinical CMV infections, signaling a distinctive set of challenges most likely based on facial mucosa. Our study outcomes underscore the imperative need for a nuanced therapeutic approach distinct from the standard triple therapy, given the disparate composition of facial and hand grafts emphasizing the necessity for a more targeted and individualized treatment regimen to optimize outcomes in both transplantations.
