# Supplementary material for: Immunosuppressive strategies in face and hand transplantation: a comprehensive systematic review of current therapy regimens and outcomes
Source: Front Transplant. 2024 Mar 6;3:1366243. doi: 10.3389/frtra.2024.1366243 (PMC11235358; doi:10.3389/frtra.2024.1366243)
Supplement: Supplementary file 1 [file Datasheet1.pdf]

Supplementary Appendix

This appendix has been provided by the authors to give readers additional information about their work.

Appendix to: **Immunosuppressive Strategies in Face and Hand Transplantation: A Comprehensive Systematic Review of Current Therapy Regimens and Outcomes;** Lioba Huelsboemer, M.D.[1±]; Sam Boroumand, B.S.[1±]; Alejandro Kochen, B.A.[1,2±]; Alna Dony, MRes.[1,3]; Jake Moscarelli, B.A.[1]; Sacha C. Hauc, B.S., B.A., MPH[1]; Viola A. Stögner, M.D.[1]; Richard N. Formica, M.D.[4]; Bohdan Pomahac, M.D.[1*]; Martin Kauke-Navarro, M.D.[1*]

[±]Contributed equally to shared first authorship
*Contributed equally to shared last authorship

Supplementary Figures and Tables:

<u>Pubmed/MEDLINE Search Terms:</u>

((((((((Vascularized Composite Allotransplantation [MeSH Terms]) OR ("Vascularized Composite Allotransplantation"[All Fields])) OR (Composite Tissue Allotransplantation [MeSH Terms])) OR ("Composite Tissue Allotransplantation"[All Fields])) OR (Composite Tissue Allografting [MeSH Terms])) OR ("Composite Tissue Allografting"[All Fields])) OR (Vascularized Composite Allograft [MeSH Terms])) OR ("Vascularized Composite Allograft"[All Fields])))

AND

(((((((("immunosuppression therapies"[All Fields]) OR (immunosuppression therapies [MeSH Terms])) OR ("antirejection therapies"[All Fields])) OR (antirejection therapies [MeSH Terms])) OR ("Immunosuppressive therapy"[All Fields])) OR (Immunosuppressive therapy [MeSH Terms])) OR ("Immunosuppression"[All Fields])) OR (Immunosuppression [MeSH Terms])))

<u>Embase Search Terms:</u>

Exp vascularized composite allotransplantation OR ("vascularized composite allotransplantation" or "composite tissue allotransplantation" or "vascularized composite allograft" or "composite tissue allografting")

AND

Exp immunosuppressive treatment OR ("immunosuppression therapies" or "antirejection therapies" or "immunosuppressive therapy" or "immunosuppression")

Supplementary Figure 1. *Full electronic search strategies for each database.*

Supplementary Table 1. *Exploration of less common immunosuppressive strategies utilized across face and hand transplantation*.

Agent	Center/Patient	Justification	Outcome
Face Induction			
Rituximab	New York, USA<br>2 Full Face	Combination approach of thymoglobulin with rituximab (chimeric monoclonal antibody against CD20- depletes mature B-lymphocytes) decreases acute rejection	Both patients remained rejection free 1 year and 3.5 years, respectively post-transplantation at time of study (2019) [61]
Hematopoietic Stem Cell Transplant	Lyon (Amiens), France<br>1 Partial Face	Received an infusion of donor bone-marrow cells to promote graft acceptance on POD 4	Developed HSV1 and EBV infection POD 185; developed posttransplant monoclonal B-cell lymphoma POM 5; developed EBV-associated posttransplant smooth muscle tumors in liver POY2; 8 acute rejection episodes across POY 2.5 [71]
Serial Extracorporeal Photochemotherapy	Lyon (Amiens), France<br>1 Partial Face	Done to avoid further progression of acute rejection process while hepatic tumor lesions were addressed	Developed HSV1 and EBV infection POD 185; developed posttransplant monoclonal B-cell lymphoma POM 5; developed EBV-associated posttransplant smooth muscle tumors in liver POY2; 8 acute rejection episodes across POY 2.5 [71]
Anti IL-2 mAB	Xi’an, China<br>1 Partial Face	Employed as quadruple therapy alongside Tacrolimus, MMF,	Developed hyperglycemia POM 1; developed pneumonia

		and Corticosteroids to reduce calmodulin inhibitor dose required and reduce toxicity of immunosuppressants and risk of infection	infection POM 1; developed 4 acute rejection episodes by POY 2 [80]
Alemtuzumab	Baltimore, USA<br>1 Full Face	Authors claim alemtuzumab provides “comparably more durable T-cell depletion with one intraoperative dose and without requirements for daily administration and monitoring” in comparison to thymoglobulin [85]	Not explicitly specified
Face Maintenance			
Azathioprine	Ankara, Turkey<br>1 Partial Face	(not specified why)	Developed CMV and bacterial infection at POM 5 and fungal infection at POM 36; developed renal dysfunction POM 5; diarrhea POM 36; vertebral osteoporosis POM 36; neutropenia POM 5; death POM 56 (sepsis) [39]
Extracorporeal Photopheresis	Paris, France<br>5 Full Face<br>2 Partial Face	Extracorporeal photopheresis can induces leucocyte apoptosis to treat/prevent acute rejection (adapted from solid organ transplantation models)	(See **Table 1** for individual patient outcomes/follow-ups)
Cyclosporine A	Saint Petersburg, Russia<br>1 Partial Face	Authors performed >40 experimental allotransplantations utilizing cyclosporine monotherapy as	Developed DIC, Anemia, Thrombocytopenia (unspecified timepoint); developed donor

		immunosuppression - adapted this approach to transplant recipient	vein thrombosis POD 1, pseudoaneurysm of donor artery POD 52; GvHD POY 2; rejections not identified [79]
Belatacept	Boston/New Haven, USA<br>1 Full Face	Switched patient to belatacept POM 14 from sirolimus due to significant lower extremity swelling, worsening renal function and rejection	Developed CMV POY 1; Guillain-Barre syndrome POM 11; 5 episodes of acute rejection across POM 24 [47]
Everolimus	Lyon (Amiens), France<br>1 Partial Face	Replaced sirolimus POY3 due to severe mouth ulcers [71]	Developed viral and fungal infections POD 185; developed post-transplant monoclonal B-cell lymphoma & hepatic EBV associated post-transplant smooth muscle tumors POM 5; 7 acute rejection episodes within POY 6
Low dose IL-2	Boston/New Haven USA<br>1 Full Face<br>1 Partial Face	Utilized to study safety and feasibility in face transplant patients	First patient experienced grade 3 rejection 17 weeks after initiation; second patient developed autoimmune hemolytic anemia [55]
Hand Induction			
Cyclophosphamide	China<br>2 Unilateral Hand	Cyclophosphamide was given with steroids and steroid cream but not specified why	Both patients developed graft loss after POY1 due to withdrawal of immunosuppression due to unmanageable pulmonary infection and chronic wound healing disorder,

			respectively; each patient had 1 acute rejection episode prior to graft loss [110]
Donor Bone Marrow Cell Infusion	Baltimore, USA<br>1 Bilateral Forearm	(not specified why)	Developed rheumatoid arthritis POY 3 [133]
Hand Maintenance			
Everolimus	Innsbruck, Austria<br>2 Bilateral Hand<br>1 Bilateral Forearm	Sirolimus or everolimus was used to replace tacrolimus/MMF as part of strategy for long-term maintenance treatment plan [101]	(See **Table 1** for individual patient outcomes/follow-ups)
Donor Bone Marrow Infusion	Pittsburgh, USA<br>1 Unilateral Hand<br>2 Bilateral Hand<br>2 Bilateral Forearm	Successfully was trialed in large animal VCA model-implemented with patient here to enable allograft survival with just low-dose tacrolimus monotherapy [142]	(See **Table 1** for individual patient outcomes/follow-ups)
